# A relict lineage and new species of green palm-pitviper (Squamata, Viperidae, *Bothriechis*) from the Chortís Highlands of Mesoamerica

**DOI:** 10.3897/zookeys.298.4834

**Published:** 2013-05-13

**Authors:** Josiah H. Townsend, Melissa Medina-Flores, Larry David Wilson, Robert C. Jadin, James D. Austin

**Affiliations:** 1 Department of Biology, Indiana University of Pennsylvania, Indiana, Pennsylvania 15705–1081, USA; 2 Centro Zamorano de Biodiversidad, Escuela Agrícola Panamericana Zamorano, Departamento de Francisco Morazán, Honduras; 3 Escuela de Biología, Universidad Nacional Autónoma de Honduras, Tegucigalpa, Francisco Morazán, Honduras; 4 Department of Ecology and Evolutionary Biology, University of Colorado Boulder, Boulder, Colorado 80309, USA; and Amphibian and Reptile Diversity Research Center, University of Texas at Arlington, Arlington, Texas 76019, USA; 5 Department of Wildlife Ecology and Conservation, University of Florida, Gainesville, Florida 32611, USA

**Keywords:** *Bothriechis guifarroi* sp. n., *Bothriechis lateralis*, *Bothriechis marchi*, Central America, conservation, cryptic species, endemic, Honduras, Pico Bonito National Park, Texíguat Wildlife Refuge

## Abstract

A new species of palm-pitviper of the genus *Bothriechis* is described from Refugio de Vida Silvestre Texíguat in northern Honduras. The new species differs from congeners by having 19 dorsal scale rows at midbody, a bright green dorsal coloration in adults, the prelacunal scale fused to the second supralabial, and in representing a northern lineage that is sister to *Bothriechis lateralis*, which is distributed in Costa Rica and western Panama and is isolated from the new taxon by the Nicaraguan Depression. This represents the 15th endemic species occurring in Refugio de Vida Silvestre Texíguat, one of the richest herpetofaunal sites in Honduras, itself being the country with the highest degree of herpetofaunal endemism in Central America. We name this new species in honor of a Honduran conservationist slain in fighting against illegal logging, highlighting the sacrifices of rural activists in battling these issues and the critical importance of conservation in these areas.

## Abstract (es)

Una nueva especie de tamagás verde del género *Bothriechis* se describe del Refugio de Vida Silvestre Texíguat en el norte de Honduras. La nueva especie difiere de sus congéneres por tener filas de 19 escamas dorsales en la mitad del cuerpo, una brillante coloración verde en la porción dorsal del cuerpo en adultos, la escama prelacunal fusionada con la segunda supralabial, y en representación del linaje del norte que es clado hermano de *Bothriechis lateralis*, la cual se distribuye en Costa Rica y el Occidente de Panamá y está aislada del nuevo taxón por la Depresión de Nicaragua. Representa la decimoquinta especie endémica encontrada en el Refugio de Vida Silvestre Texíguat, uno de los lugares más ricos de herpetofauna en Honduras, el país con el más alto grado de endemismo de la herpetofauna en Centroamérica. Nombramos esta nueva especie en honor a un conservacionista Hondureño asesinado en contienda contra la tala ilegal, destacando los sacrificios de los activistas rurales luchando frente a estos problemas y la importancia crítica de la conservación en estas áreas.

## Introduction

In the past decade, a steady stream of taxonomic discoveries have come out of the Chortís Highlands of Mesoamerica, a biogeographic region found to the south and east of the tectonic boundary between the Chortís and Mayan Blocks and north of the Nicaraguan Depression (Townsend 2011, Townsend et al. 2011). Fifty new species of amphibians and reptiles have been described from the region’s montane forests since 2000 (Cadle 2012, Cadle and Savage 2012, McCranie and Hedges 2012, Rovito et al. 2012), with literally dozens more awaiting description (Townsend 2011).

Our knowledge of the taxonomic diversity of Mesoamerican pitvipers has also greatly increased since the turn of the century (e.g. Campbell and Flores-Villela 2008, Jadin et al. 2011). Three species of endemic pitvipers have been described from the Chortís Highlands since 2000: *Atropoides indomitus* Smith & Ferrari-Castro 2008, *Bothriechis thalassinus* Campbell & Smith 2000, and *Cerrophidion wilsoni* Jadin, Townsend, Castoe & Campbell 2012. Two of these three taxa, *Bothriechis thalassinus* and *Cerrophidion wilsoni*, had previously been concealed within more widespread taxa only to be revealed by more focused sampling and phylogenetic analyses.

The green palm-pitvipers (genus *Bothriechis*) of Mesoamerica have long been a source of taxonomic uncertainty and confusion (Campbell and Lamar 2004). Ambiguities among type specimens and localities, the imprecise provenance of many available specimens, disjunct distributions limited to fragmented highland forests, and misleading external morphology have all contributed to a lack of taxonomic resolution among populations currently assigned to two species from the Chortís Highlands; *Bothriechis marchi* (Barbour and Loveridge 1929) and *Bothriechis thalassinus*. As currently understood, these two taxa inhabit a number of disjunct localities in the Chortís Highlands (Campbell and Lamar 2004, McCranie 2011a). Available molecular data for these two taxa are limited to a single sample assigned to each nominal form, which indicate the two are sister species nested within a Nuclear Central American clade (also including *Bothriechis aurifer*, *Bothriechis bicolor*,and *Bothriechis rowleyi*) that is, in turn, sister to the highland *Bothriechis* (i.e. *Bothriechis lateralis* and *Bothriechis nigroviridis*) found in lower Central America (Taggart et al. 2001, Castoe and Parkinson 2006, Castoe et al. 2009).

*Bothriechis marchi* *sensu lato* is known from localities in the Cordillera Nombre de Dios, Cordillera de Merendón, and Sierra de Sulaco of Honduras and adjacent areas of Guatemala, with *Bothriechis thalassinus* being found in the Cordillera de Merendón of Guatemala and Honduras, Cerro Santa Bárbara and nearby highland forests in Honduras, Cerro del Mono in eastern Guatemala, and the highlands around the El Salvador-Guatemala-Honduras border area (Campbell and Lamar 2004; McCranie 2011a). Both of these taxa include a number of allopatric highland populations that have not been assessed using phylogenetic methods. Of interest here are populations from the Cordillera Nombre de Dios, found within and around Refugio de Vida Silvestre Texíguat and Parque Nacional Pico Bonito. These two cloud forest reserves are each recognized for their diverse endemic herpetofauna (McCranie and Castañeda 2005; Townsend et al. 2012) and are taxonomically and biogeographically distinctive from that of the northern Cordillera de Merendón, which includes the vicinity of the type locality of *Bothriechis marchi* in the Sierra de Omoa (Townsend and Wilson 2008).

Two expeditions in 2010 provided the first herpetofaunal inventory of the extensively forested windward portions of Refugio de Vida Silvestre Texíguat, one of the most endemism-rich highland forests in Mesoamerica (Townsend et al. 2012). During two visits to the windward side of Refugio de Vida Silvestre Texíguat in June and July 2010, we collected a series of arboreal pitvipers representative of those assigned to *Bothriechis marchi*. Phylogenetic analyses revealed that the population from Refugio de Vida Silvestre Texíguat is not conspecific with the nominal taxon *Bothriechis marchi*, nor are they part of the Nuclear Central American clade containing *Bothriechis marchi* and *Bothriechis thalassinus*. Remarkably, this population is shown to represent a relict northern lineage that is most closely related to *Bothriechis lateralis* from Costa Rica and western Panama. We herein describe the Refugio de Vida Silvestre Texíguat population of *Bothriechis* as a new taxon, and discuss the implications for systematics, biogeography, and conservation.

## Materials and methods

### Field-based sampling

The type series was collected during sampling in the vicinity of La Liberación (15.53°N, 87.29°W; camp established at 1,030 m elevation) during 10–21 June (11 participants; 1,320 person-hours sampling) and 26 July–2 August 2010 (13 participants; 880 person hours). Tissue samples were preserved in SED buffer (20% DMSO, 0.25 M EDTA, pH 7.5, NaCl saturated; Seutin et al. 1991, Williams 2007) and whole specimens in 10% formalin and later transferred to 70% ethanol. Specimens were deposited in the Carnegie Museum of Natural History (CM), Museum of Vertebrate Zoology, University of California Berkeley (MVZ), National Museum of Natural History, Smithsonian Institution (USNM), and Amphibian and Reptile Diversity Research Center, University of Texas at Arlington (UTA).

### DNA extraction, amplification, and sequencing

Genomic DNA was isolated from muscle tissue taken from eleven specimens of *Bothriechis* using a Qiagen DNeasy extraction kit and protocol. Four mitochondrial gene fragments (NADH dehydrogenase subunit 4 (ND4), cytochrome b (cyt *b*), 12S rRNA, and 16S rRNA) were independently PCR-amplified as described in multiple studies (Knight and Mindell 1993; Arévalo et al. 1994; Parkinson et al. 1997; Parkinson et al. 2002) using Promega GoTaq® Green master mix, the primer pairs ND4 + LEU, Gludg + AtrCB3, L1091 + 12E, and 16SF + 16SR, and annealing temperatures 48°C, 48°C, 50°C, and 45°C, respectively. Sequencing was performed in both forward and reverse directions using the PCR primers on a Beckman Coulter automated capillary sequencer, and sequence chromatographs were edited using Sequencher 4.2. Sequences for each gene were aligned separately, first automatically using the program MUSCLE (Edgar 2004), and then manually rechecked using Se-Al v2.0a11. Gaps in alignments were treated as missing data. No internal stop codons were found in the two protein-coding gene fragments. Novel sequences from this study were deposited in GenBank (KC847255–289).

Previously published sequences of *Bothriechis* were downloaded from GenBank and combined with new sequence data generated in this study (Table 1). Representatives of two Mesoamerican genera from the diverse sister clade to *Bothriechis* (which contains *Atropoides* [Mesoamerica], *Bothriopsis* [South America], *Bothrocophias* [South America], *Bothrops* [Mexico to South America], *Cerrophidion* [Mesoamerica], and *Porthidium* [Mexico to South America]) were selected for use as outgroups to root our *Bothriechis* phylogeny (Castoe et al. 2005, 2009; Daza et al. 2010): *Atropoides mexicanus* and *Cerrophidion wilsoni*, both of which are sympatric with *Bothriechis* in Refugio de Vida Silvestre Texíguat (Townsend et al. 2012).

**Table 1.** Taxa, vouchers, locality data, and GenBank accession numbers for sequences used in this study. Novel sequences from this study are indicated in boldface; country codes used as follows: CR = Costa Rica; EC = Ecuador; GT = Guatemala; HN = Honduras; MX = Mexico; NI = Nicaragua.<br/>

**Taxon**	**Locality**	**Voucher**	**GenBank Accession Numbers**			
			**ND4**	**cyt *b***	**12S**	**16S**
*Atropoides mexicanus*	HN: Atlántida: Texíguat	USNM 578906	**KC847289**	**KC847271**	**KC847268**	**KC847255**
*Bothriechis aurifer*	GT:	UTA R-35031	DQ305483	DQ305466	DQ305425	DQ305448
*Bothriechis bicolor*	GT:	UTA R-34156	DQ305484	DQ305467	DQ305426	DQ305449
*Bothriechis guifarroi*	HN: Atlántida: Texíguat	CM 156870	—	**KC847280**	—	**KC847260**
	HN: Atlántida: Texíguat	MVZ 269305	—	**KC847279**	—	**KC847258**
	HN: Atlántida: Texíguat	USNM 579873	**KC847286**	**KC847274**	**KC847267**	**KC847262**
	HN: Atlántida: Texíguat	USNM 579874	**KC847288**	**KC847282**	**KC847266**	**KC847263**
	HN: Atlántida: Texíguat	USNM 579875	**KC847287**	**KC847281**	**KC847265**	**KC847264**
	HN: Atlántida: Texíguat	USNM 579876	—	**KC847276**	—	—
	HN: Atlántida: Texíguat	USNM 579877	—	**KC847278**	—	—
	HN: Atlántida: Texíguat	USNM 579878	—	**KC847277**	—	**KC847261**
	HN: Atlántida: Texíguat	UTA R-60303	—	**KC847275**	—	**KC847259**
*Bothriechis lateralis*	CR: Acosta	MZUCR-11155	U41873	AY223588	AF057211	AF057258
*Bothriechis marchi*	GT: Zacapa: Cerro del Mono	UTA R-52959	DQ305486	DQ305469	DQ305428	DQ305451
	HN: Cortés: Sierra de Omoa	MVZ 263604	—	**KC847283**	—	—
*Bothriechis nigroviridis*	CR: San Gerondo de Dota	MZUCR-11151	AY223635	AY223589	AF057212	AF057259
*Bothriechis rowleyi*	MX: Cerro Baúl	JAC 13295	DQ305485	DQ305468	DQ305427	DQ305450
*Bothriechis schlegelii*	CR: Cariblanco de Sarapiquí	MZUCR-11149	AY223636	AY223590	AF057213	AF057260
	EC: Pichincha	FHGO Live coll	AF292611	AF292573	—	—
	HN: Cortés: Yojoa	UF 157577	**KC847285**	**KC847272**	**KC847270**	**KC847257**
	NI: Jinotega: Bosawas	UF 166874	**KC847284**	**KC847273**	**KC847269**	**KC847256**
*Bothriechis supercilliaris*	CR: San Vito	—	DQ305487	DQ305470	DQ305429	DQ305452
*Bothriechis thalassinus*	GT: Zacapa	UTA R-52958	DQ305482	DQ305465	DQ305424	DQ305447
*Cerrophidion wilsoni*	HN: Olancho: Botaderos	UTA R-52953	JQ724172	JQ724159	JQ724146	JQ627132

### Phylogenetic analyses

Bayesian inference (BI) and maximum likelihood (ML) were implemented to reconstruct phylogenies for the *Bothriechis* ingroup taxa. To identify appropriate models of nucleotide substitution for both analyses, we used the program MrModeltest v2.2 (Nylander 2004), run in PAUP* v4.0b10 (Swofford 2002). We used Akaike information criterion (AIC) to select the best-fit models, as estimated by MrModeltest (Table 2). The four gene fragments were concatenated (2,263 total bp), and this combined dataset was partitioned by gene and codon position (for cyt-b and ND4), resulting in a total of eight partitions as was shown to be justified in analysis of a similar dataset that included these four fragments from these species (Castoe and Parkinson 2006). Stems and loops were not partitioned separately due to a lack of informative characters.

Phylogenetic analyses using BI were conducted with MrBayes v3.0b4 (Ronquist and Huelsenbeck 2003). Two simultaneous BI runs were conducted (with the default Markov chain Monte Carlo [MCMC] settings), and run for a total of 5.0 × 10^6^ generations per run, sampling trees and parameters every 100 generations. We used PSRF values (output by MrBayes), together with plots of cold chain likelihood values and parameter estimates visualized in Tracer v1.5.4 (Rambaut and Drummond 2009), to confirm stationarity and convergence of MCMC runs. Based on this evaluation, the first 1.5 × 10^5^ generations from each run were discarded as burn-in.

Phylogenetic relationships were inferred using ML as implemented in RAxML 7.2.8 (Stamatakis 2006, Stamatakis et al. 2008), using the same partitioning scheme described above for BI. Tree support was assessed using the rapid-bootstrapping algorithm with 1000 non-parametric bootstraps; all ML estimates and tests were run under the GTRCAT model, as models available for use in RAxML are limited to variations of the general time-reversible (GTR) model of nucleotide substitution.

**Table 2.** Results from *a priori* model selections based on *Akaike* information criterion (AIC) conducted in MrModeltest 2.2 (Nylander, 2004) for partitions of the dataset.<br/>

**Partition**	**Total characters**	**Parsimony-informative characters**	**Best-fit model**
ND4 1^st^ pos	222	39	GTR+Γ
ND4 2^nd^ pos	222	9	GTR+I
ND4 3^rd^ pos	222	128	GTR+Γ
Cyt *b* 1^st^ pos	231	33	GTR+Γ
Cyt *b* 2^nd^ pos	231	12	HKY+I
Cyt *b* 3^rd^ pos	231	134	GTR+I+Γ
12S	409	60	GTR+Γ
16S	495	39	GTR+I

### Morphological data collection

We examined 34 preserved specimens of *Bothriechis* for this study (Appendix). Definitions of scale counts and morphological features follow Campbell and Lamar (2004) and bilateral characters are reported as right/left. Unsexed juvenile specimens were considered separately from adult males and females. For the holotype, we dissected and removed the partially everted hemipenis at the base. We then filled the hemipenis with warm water using a blunt-tipped syringe needle in order to attempt full eversion. We then removed the water and injected hot petroleum jelly with blue wax-dye until near maximum expansion was achieved. Finally, we tied the hemipenes and stored them in 70% ethanol. This procedure is modified from that of Myers and Cadle (2003) and Zaher and Prudente (2003) and is further described and illustrated in Smith and Ferrari-Castro (2008) and Jadin and Smith (2010). Hemipenial terminology follows Dowling and Savage (1960), Keogh (1999), and Savage (2002). Comparative morphological data on related species was taken primarily from Campbell and Lamar (2004) and Solórzano (2004). Color names and codes used in descriptions of coloration in life are from Köhler (2012); color notes in life were derived from a series of photographs of the holotype and paratypes.

## Results

**Figure 1.**
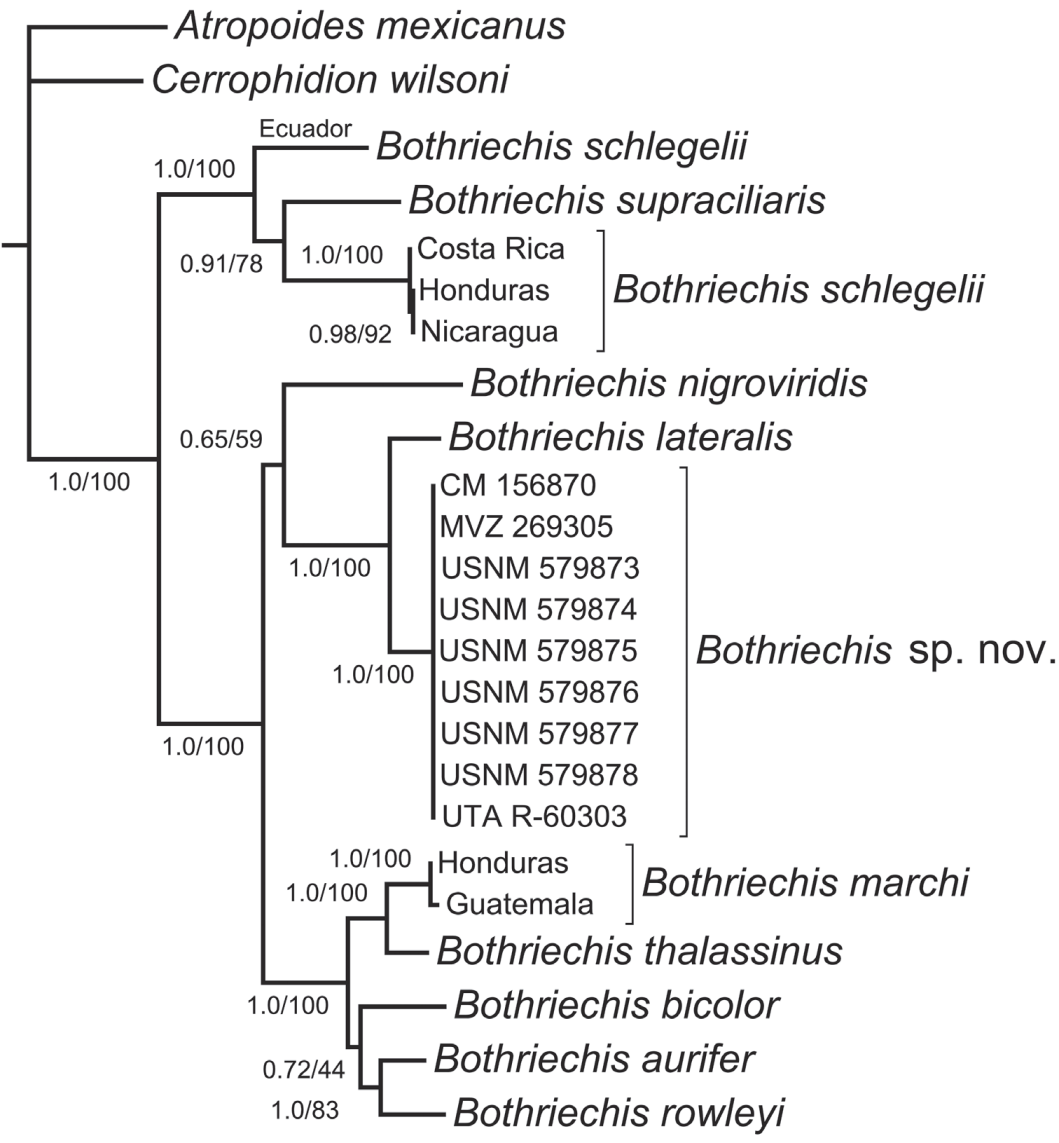
Phylogeny of palm-pitvipers (genus *Bothriechis*), showing strong support for a species-level clade of the Texíguat population (*Bothriechis* sp. n.) that is sister to *Bothriechis lateralis*. The tree was estimated from a Bayesian 50% majority-rule consensus composed from a concatenated mitochondrial dataset (ND4, cyt *b*, 12S, and 16S; total of 2263 bp). Numbers at nodes represent values of Bayesian posterior probabilities (PP, left) and Maximum Likelihood bootstraps (BS, right). Nodes supported by ≥ 95% PP and ≥ 70 BS are considered highly supported.

Bayesian and Maximum Likelihood phylogenetic analyses produced congruent topological results. Our phylogeny (Fig. 1) is generally congruent with those of Castoe et al. (2009) and Daza et al. (2010), recovering two clades of nominal *Bothriechis schlegelii* (one Mesoamerican, one from Ecuador) rendered paraphyletic with respect to *Bothriechis supraciliaris*, and showing strong support for a *Bothriechis marchi*–*Bothriechis thalassinus* clade and a *Bothriechis aurifer*–*Bothriechis rowleyi* clade, together forming a *Bothriechis aurifer*–*Bothriechis bicolor*–*Bothriechis marchi*–*Bothriechis rowleyi*–*Bothriechis thalassinus* clade that geographically corresponds to Nuclear Central America (Fig. 1). Both of our analyses recovered a weakly supported clade that includes the Costa Rica/Panama taxa *Bothriechis lateralis* and *Bothriechis nigroviridis*, along with the *Bothriechis* population from Refugio de Vida Silvestre Texíguat, Honduras. Within this primarily southern clade, nine samples from Refugio de Vida Silvestre Texíguat show virtually no genetic divergence from one another, and form a monophyletic group with a well-supported (PP = 1.0; bs = 100) sister clade to *Bothriechis lateralis* from Costa Rica and western Panama (Fig. 1).

Based on the phylogenetic results, we examined morphological variation among populations of *Bothriechis marchi* *sensu lato*, which confirm the evolutionary and taxonomic distinctiveness of the Texíguat population as well as its apparent morphological affinity with a specimen from Parque Nacional Pico Bonito, approximately 75 km to the east of Refugio de Vida Silvestre Texíguat. We present the following description of this relict northern lineage as a new species.

## Systematics

### Bothriechis guifarroi sp. n.

urn:lsid:zoobank.org:act:1FC0661F-B08B-4D0D-85CC-DAEAB502D0DB

http://species-id.net/wiki/Bothriechis_guifarroi

Figs 2 –3 , 5 –6

Bothrops nigroviridis (in part): Meyer 1969: 420.
Bothriechis marchi (in part): Campbell 1982: 381.
Bothrops marchii (in part):Wilson and Meyer 1985: 120.

#### Holotype.

**Figure 2.**
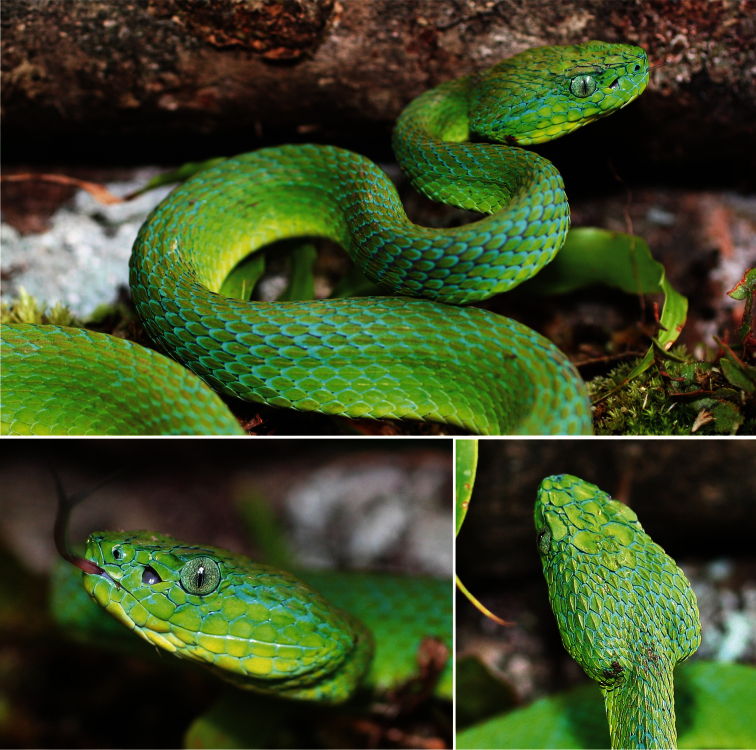
Photographs in life of the adult male holotype of *Bothriechis guifarroi* (UTA R-60303), with lateral and dorsal views of the head. Photographs by JHT.

**Figure 3.**
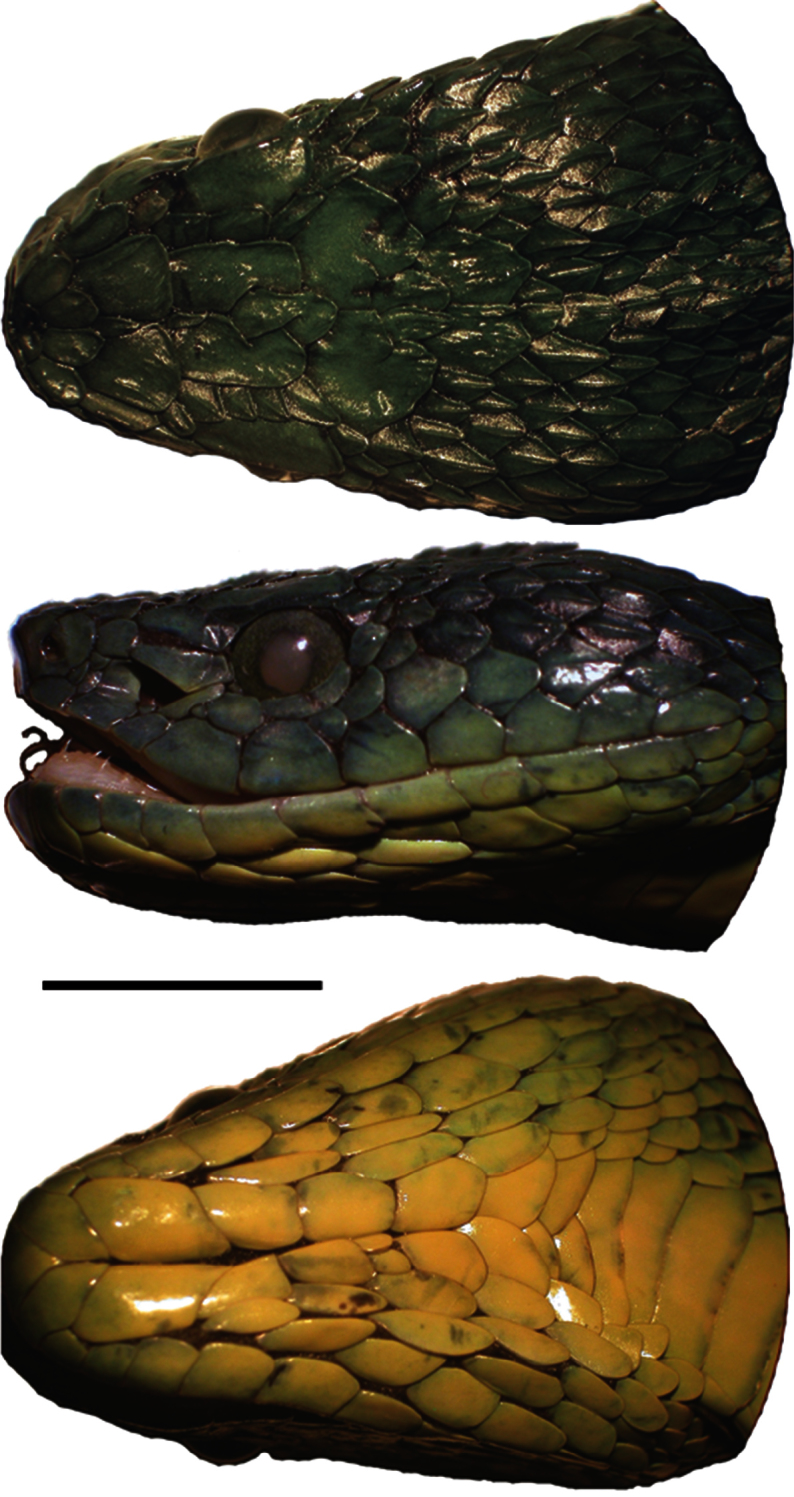
Dorsal, lateral, and ventral aspects of the head of the holotype of *Bothriechis guifarroi* (UTA R-60303). Photographs by RCJ.

**Figure 4.**
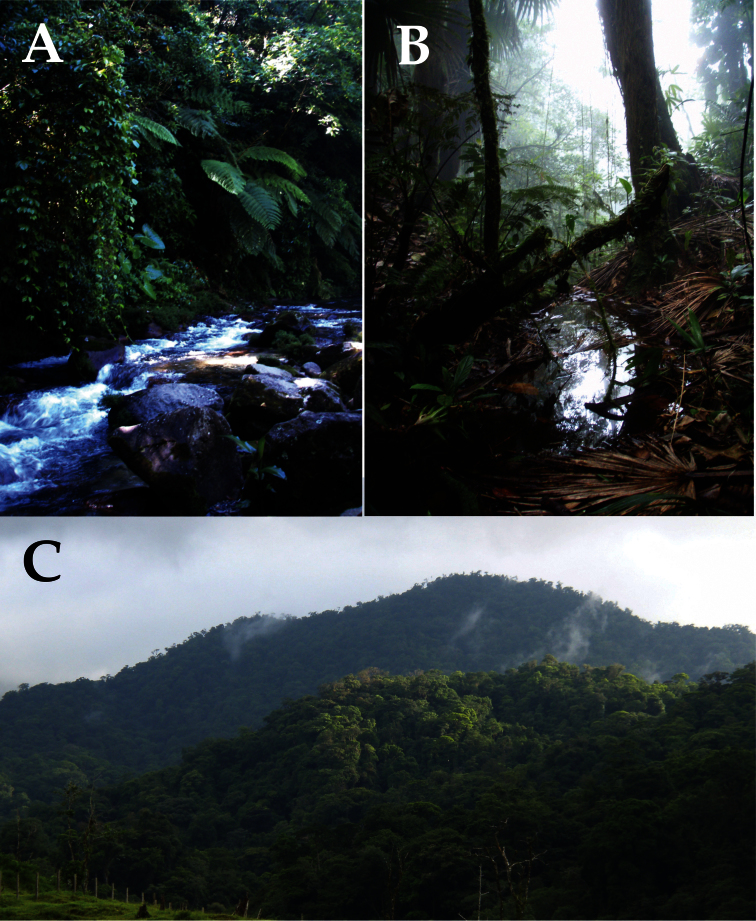
Habitat in the vicinity of the type locality of *Bothriechis guifarroi*,La Liberación, Refugio de Vida Silvestre Texíguat, Honduras; **A** riparian vegetation along the Río Jilamito, 1,015 m elevation **B** small seepage pond near the top of Cerro El Chino, 1,380 m elevation **C** premontane rainforest with the clearing aroundLa Liberación (1,030 m elevation) visible in the foreground. Photographs by JHT.

UTA R-60303 (Figs 2, 3), an adult male from La Liberacíon (Fig. 4A, C), 15.5302°N, 87.2939°W (DD), 1,015 m elevation, Refugio de Vida Silvestre Texíguat, Departamento de Atlántida, Honduras, collected 25 July 2010 by the field team of E. Aguilar, A. Contreras, L. Gray, L.A. Herrera-B., M. Medina-Flores, A. Portillo, A. Stubbs, and J. H. Townsend. Original field number JHT 3243. Genbank accession numbers: 16S (KC847259), cyt *b* (KC847275).

#### Paratypes (8):

HONDURAS: Departamento de Atlántida: Refugio de Vida Silvestre Texíguat: adult female (USNM 579875) collected 19 June 2010, two adult females (USNM 579876–77) collected 29 July 2010, and two unsexed neonates collected 18 June 2010 (USNM 579873–74), all from Cerro El Chino (Fig. 4B), 15.5225°N, 87.2802°W (DD), 1,360–1,450 m elevation, southeast of La Liberación. Two males (CM 156870 and MVZ 269305) collected 28 July 2010 from a ridge-top trail above La Liberación, 15.5418°N, 87.2891°W (DD), 1,290 m elevation. One male (USNM 579878) collected 30 July 2010 from La Liberación (Fig. 4A, C), 15.5302°N, 87.2939°W (DD), 1,015 m elevation.

#### Referred specimens (4).

HONDURAS: Departamento de Atlántida: AMNH 46949 from “Tela,” collected sometime before April 1932; USNM 319942 from Quebrada de Oro, Parque Nacional Pico Bonito. Departamento de Yoro: Refugio de Vida Silvestre Texíguat: USNM 337488–89 from 2.5 airline km north-northeast of La Fortuna. See Remarks.

#### Definition.

*Bothriechis guifarroi* is distinguished from all nine congeners by the following combination of features: dorsal scales in 19-19-15 rows; ventrals in males 162–166 (163.8), in females 158–166 (164.0), 162–166 (164.0) in neonates; subcaudals in males 60–68 (63.0), in females 60–63 (61.0), 62–68 (65.0) in neonates; intersupraoculars (3–7); superciliary scales absent; prelacunal scale fused to second supralabial on both sides; two known color patterns in juveniles, one brown (with a pale paraventral stripe and a series of short darker dorsal blotches and a dark brown postocular stripe bordered by yellow on its lower edge) and the other green (with a series of pale blue blotches and a deep blue postocular stripe bordered by pale blue on its lower edge); dorsal coloration in adults green with pale blue trim on anterior edges of dorsal scales, and pale blue postocular stripe with green along the keels in center of stripe; and iris pale green, pale gray, or pale tan.

#### Diagnosis.

*Bothriechis guifarroi* can be distinguished from the other members of the genus *Bothriechis* as follows (*Bothriechis guifarroi* features indicated first, those for species compared next): *Bothriechis aurifer* (distributed at moderate and intermediate elevations from extreme east-central Chiapas, Mexico, to east-central Guatemala) — adult color pattern (green vs. black-bordered yellow blotches on green background and prominent black postocular stripe) and juvenile color pattern (green with pale blue blotches or brown with pale paraventral stripe and dark dorsal blotches vs. pale lime green with black-bordered yellow blotches); *Bothriechis bicolor* (occurring marginally at low upward to intermediate elevations from southeastern Chiapas, Mexico, to south-central Guatemala) — number of dorsal scales at midbody (19 vs. 21) and condition of prelacunal and second supralabial scales (fused vs. separate); *Bothriechis lateralis* (moderate to marginally high elevations from northwestern Costa Rica to western Panama) — number of dorsal scale rows at midbody (19 vs. modal number of 23), adult color pattern (green vs. green with pale paravertebral bars and paraventral stripe), and juvenile color pattern (bi-morph pattern of green with blue dorsal blotching or brown with pale paraventral stripe and short unicolor dark blotches vs. uni-morph pattern of brown ground color with pale paraventral stripe and short bicolor dark and pale blotches); *Bothriechis marchi* (found marginally at low elevations up to intermediate elevations in northwestern Honduras and adjacent Guatemala) — condition of prelacunal and second supralabial scales (fused vs. separate), number of subcaudals in females (60–63 vs. 46–57); *Bothriechis nigroviridis* (moderate to intermediate elevations from north-central Costa Rica to west-central Panama) — adult color pattern (patternless green vs. green with very heavy black mottling), juvenile color pattern (green with pale blue blotches or brown with pale paraventral stripe and a series of short darker dorsal blotches vs. green with heavy black mottling), iris color (pale green, pale gray, or pale tan vs. almost black), numbers of ventral scales in both sexes (162–166 and 158–166 vs. 143–158 and 134–158), numbers of subcaudal in both sexes (60–68 and 60–63 vs. 49–56 and 44–58), and condition of prelacunal and second supralabial scales (fused vs. separate); *Bothriechis rowleyi* (moderate to intermediate elevations from extreme southeastern Oaxaca to northwestern Chiapas, Mexico) — condition of prelacunal and second supralabial scales (fused vs. almost always separate), iris color (pale green, pale gray, or pale tan vs. yellow), and juvenile color pattern (green with pale blue blotches or brown with pale paraventral stripe and a series of short darker dorsal blotches vs. pale yellowish green with brown or purple dorsal blotches); *Bothriechis schlegelii* (low to intermediate elevations from northwestern Chiapas, Mexico, southward through Central America and into northwestern South America as far as extreme western Venezuela and extreme northern Peru) — lack of superciliary scales in the former and their presence in the latter, number of supralabials (10–12, usually 10 vs. 7–10, usually 8), number of midbody dorsal scale rows (19 vs. 21–25, usually 23), and adult color pattern (green vs. extremely variable color and pattern involving ground color of yellow, pink, brown, gray, or green and dorsal blotching of a sizable array of colors, but sometimes absent; contrasting postocular stripe absent vs. present); *Bothriechis supraciliaris* (moderate to intermediate elevations from southwestern Costa Rica to west-central Panama) — lack of superciliary scales in the former and their presence in the latter, number of midbody dorsal scale rows (19 vs. 21–23, usually 23), number of ventral scales in both sexes (162–166 and 158–166 vs. 145–150 and 141–148), number of subcaudal scales in both sexes (60–68 and 60–63 vs. 48–54 and 45–52), and adult color pattern (green vs. extremely variable color and pattern involving ground color of shades of green, brown, or maroon and dorsal blotching of an array of colors contrasting with that of the ground color; contrasting postocular stripe absent vs. present); *Bothriechis thalassinus* (moderate to intermediate elevations from extreme eastern Guatemala and extreme northwestern El Salvador to western Honduras) — number of midbody dorsal scale rows (19 vs. 21–23, usually 21), and condition of prelacunal and second supralabial scales (fused vs. separate).

#### Description of holotype.

An adult male (Figs 2, 3) with hemipenes partially everted, left removed;rostral broader than high (4.38 × 3.26 mm); 2 internasals anteriorly; 2/2 canthals; 4 posterior intercanthals; supraoculars slightly more than two times as long as broad; 5 intersupraoculars; many scales on head of large size, including large, flat frontal and parietal scales; interrictals 25; single loreal, longer than high, bounded by upper two preoculars, canthal above, prelacunal and prefoveals below, and nasal; prefoveals 3/3, subfoveals 1/1; prelacunal and second supralabial fused; preoculars 3/3, upper largest, middle large and in contact with supralacunal; suboculars 2/2; postoculars 2/2; supralabials 10/10; mental broader than long (4.39 x 3.29 mm); infralabials 11/11; chin shields contacting first four pairs of infralabials; gulars between chin shields and first preventral 6/4; dorsal scale rows 19-19-15; preventrals 2; ventrals 161; cloacal scute undivided; 65 undivided subcaudals; tail spine short and blunt.

**Figure 5.**
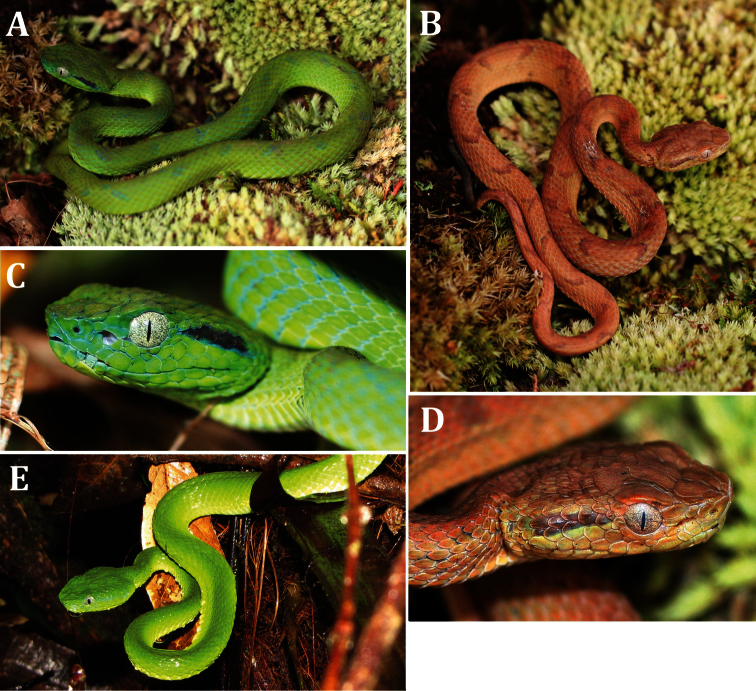
Paratypes of *Bothriechis guifarroi* in life; **A** USNM 579874, green-phase juvenile **B** USNM 579873, brown-phase juvenile **C** close-up of head of USNM 579874 **D** close-up of head of USNM 579873 **E** USNM 579875, female paratype photographed in situ at Cerro El Chino, 1,420 m elevation. Photographs by JHT.

**Figure 6.**
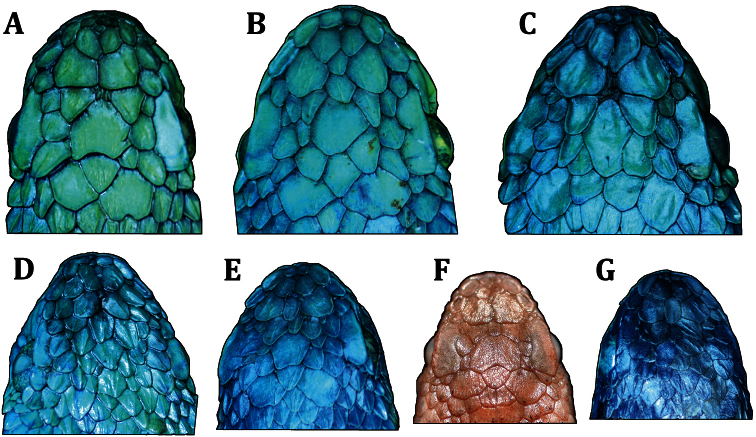
Variation in dorsal head scales among paratypes of *Bothriechis guifarroi*; **A** USNM 579877, adult female **B** MVZ 269305, adult male **C** USNM 579876, adult female **D** USNM 579875, adult female **E** USNM 579878, adult male **F** USNM 579873, neonate **G** USNM 579874, neonate. Photographs by JHT.

#### Measurement of holotype.

Total length 734 mm; tail length 136 mm, comprising 18.5% of total length; head 29.8 mm from front face of rostral to posterior end of mandible; head 19 mm at broadest point; neck 7 mm directly behind jaws.

#### Hemipenis description of holotype.

The partially everted left hemipenis of the holotype is described. Hemipenis at least 20 mm in total length and 13 mm in maximum width at level of crotch; on sulcate side base with several rows of small spines (< 0.5 mm) followed by rows of larger spines and hooks extending for 5 mm, largest protruding ca. 3.5 mm; asulcate side with minutely spined base up to 7 mm before level of bilobation; numerous small mesial spines (< 0.5 mm) arranged in rows present for 4 mm to the calyces, with peripheral section of each lobe containing nine spines and hooks (≥ 2 mm), five of which border lower rim of calyces; calyces follow spines and hooks distally; calyces scalloped, at least 10 rows at least 7 mm to apex of hemipenis on asulcate side; sulcus spermaticus deep and bifurcating ca. 4 mm before site of bilobation and extending upwards through spines and calyces likely to tip of each lobe; sulcus spermaticus bordered by two, occasionally three, columns of minute scales to the beginning of calyces, which form the border likely to the apex of the lobes. Although the majority of the hemipenial characters of this specimen are reported, the lack of a fully everted hemipenis leaves information on the total length and the nature of the calyces incomplete.

#### Coloration of holotype in life.

Middorsal scales of the holotype Yellowish Spectrum Green (Color 128), fading to Light Grass Green (Color 109) laterally and becoming Chartreuse (Color 89) ventrolaterally, with Medium Greenish Yellow (Color 88) ventral scales; dorsal body scales edged anteriorly in Light Caribbean Blue (Color 163), with up to approximately one-fourth of the anterior end of some scales edged in blue; skin concealed between dorsal scales Spectrum Violet (Color 186); postoccipital stripe Light Caribbean Blue (Color 163), with keel and adjacent portion of three scales that lie within the postoccipital stripe Light Emerald Green (Color 142); terminal portion of tail Plumbeous (Color 295); iris Pale Bluish Gray (Color 287) with fine black reticulations most heavily concentrated around the pupil.

#### Color pattern of holotype in preservative.

Scales on dorsal surfaces of the head and body blue-green, becoming more green laterally and yellow-green to yellow ventrally. Tail is mostly green with some grayish blue-green at the dorsal base. Pupil is cloudy and pale, surrounded by lime green iris heavily speckled with black.

#### Variation in paratypes.

We discuss scutellational variation in the three adult male paratypes first, the three adult females next, and finally the two unsexed neonates. Scutellation varies as follows (range followed by mean): ventrals (162–166 [164.3], 158–166 [161.7], 162 and 166); subcaudals (60–68 [64.0], 60–63 [61.0], 62 and 68); ventrals + subcaudals (222–233 [228.3], 221–226 [222.7], 228 and 230); cloacal scute entire in all specimens; dorsal scale row formula 19-19-15, with the reduction to 15 rows occurring at ventrals 114–162; supralabials (10–11 [10.2], 10—11 [10.2], 10–10 and 12–11); infralabials (10–12 [11.0], 10–13 [11.7], 11–11 and 12–11); preoculars 2–2 in all specimens, except 3-3 in CM 156870; postoculars 2–2 in all specimens, except 3–2 in MVZ 269305 and 4–4 in CM 156870; suboculars 2–3 [2.5], 2–3 [2.7], 2–2 and 3–4; relative tail length (0.184–0.223 [0.199], 0. 167–0.182 [0.176], 0.179 and 0.194).

Two juvenile color patterns are present in this species (Fig. 5), one we refer to as a “green phase”, the other as a “brown phase.” Both juvenile phases have distinctively colored tail-tips, presumably used in caudal luring, and well-differentiated postocular stripes. The green phase (USNM 579874) has a Chartreuse (Color 89) dorsal ground color with Light Turquoise Green (Color 146) edging on the dorsal scales as well as on a series of irregular middorsal blotches, a Pale Green (Color 99) venter, and a Chartreuse (Color 89) head with a Jet Black (Color 300) postocular stripe, bordered above and below by Light Turquoise Green (Color 146); tip of tail Cobalt Blue (Color 180); the iris is Pale Neutral Gray (Color 296) with fine darker reticulations. The brown phase (USNM 579873) has a Robin Rufous (Color 29) ground color middorsally and anteriorly, becoming Salmon Color (Color 58) laterally and posteriorly, with a series of irregular Ferrunginous (Color 35) middorsal blotches, a Pale Buff (Color 1) ventral surface of head and venter becoming gradually darker (Pale Pinkish Buff [Color 3]) posteriorly, a Dark Salmon Color (Color 59) head with a Chestnut (Color 30) postocular stripe, bordered above and below by Light Buff (Color 2); tip of tail Sepia (Color 286); a Pale Buff (Color 1) paraventral stripe is present on the lower half of the first dorsal scale row and the lateral edge of the ventrals; dark speckling along lateral edge of the ventrals; the iris is Chamois (Color 84) with fine darker reticulations.

The type series of *Bothriechis guifarroi* demonstrates considerable variation in the condition and shape of the scales on the dorsal surface of the head (Fig. 6), a characteristic often considered diagnostic among *Bothriechis* (Campbell and Smith 2000, McCranie 2011a). Two adult females (USNM 579875 [Fig. 6C] and USNM 579877 [Fig. 6A]), one adult male (MVZ 269305 [Fig. 6B]), and one neonate (USNM 579873 [Fig. 6F]) all have multiple enlarged, unkeeled, plate-like scales present anterior to the posteriormost edge of the orbits; one adult female ([Fig. 6D]), one adult male ([Fig. 6E]), and one neonate (Fig. 6G) all have smaller keeled scales present anterior to the posteriormost edge of the orbits. As a result of demonstrating essentially the full range of dorsal head scale conditions in the type series, we do not consider this characteristic to be of value in diagnosing *Bothriechis guifarroi* from other congeners.

#### Etymology.

The specific name *guifarroi* is a patronym used to honor our colleague and friend, Honduran environmental leader Mario Guifarro of Olancho. Don Mario fearlessly led grassroots efforts to stop illegal logging in the indigenous Tawahka territory of eastern Honduras, despite repeated assassination attempts and threats on his own life and those of his compatriots. Don Mario was murdered on 15 September 2007, ironically Honduras’ Independence Day, while leading a mission to demarcate the boundaries of the Tawahka-Asangni Biosphere and stave off further illegal deforestation. On 21 July 2008, the only witness to Mario’s assassination, his son Shamir Guifarro Ramírez, was also murdered, along with Mario’s father-in-law, Henry Arturo Chacón, and mother-in-law, Nelda Ochoa, after they were followed out of the city of Juticalpa by unknown assailants.

#### Distribution.

**Figure 7.**
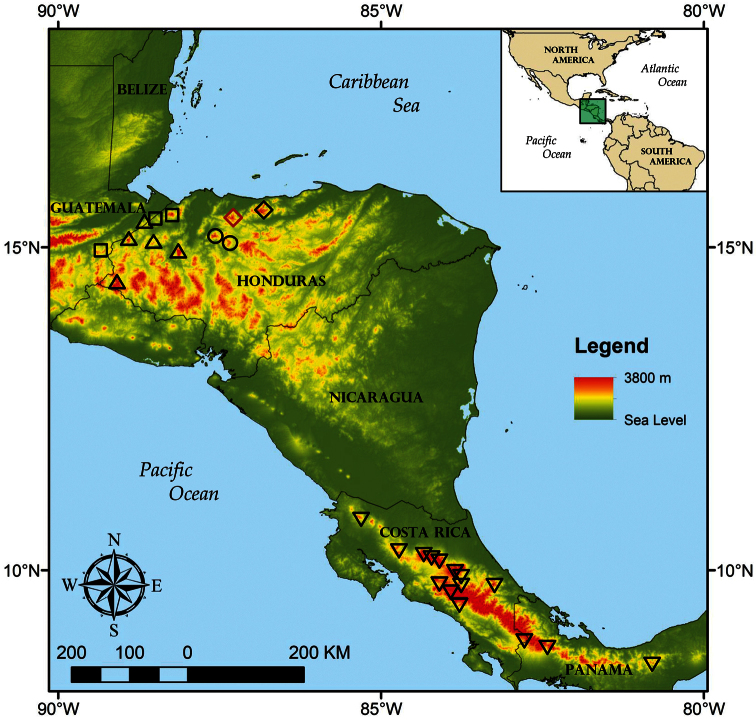
Geographic distribution of selected *Bothriechis* species and populations discussed in the text; localities are based on data published herein and those of Campbell and Lamar (2004), McCranie (2011a), and Savage (2002); red diamond = type locality of *Bothriechis guifarroi* in Refugio de Vida Silvestre Texíguat; black diamond = referred population of *Bothriechis guifarroi* from Parque Nacional Pico Bonito; circles = *Bothriechis* sp. *inquirenda* populations for the Sierra de Sulaco; squares = *Bothriechis marchi*; triangles = *Bothriechis thalassinus*, inverted triangles = *Bothriechis lateralis*.

Populations genetically confirmed to represent *Bothriechis guifarroi* are found between 1,015–1,450 m elevation in the western portion of the Cordillera Nombre de Dios, Department of Atlántida, Honduras, within the boundaries of Refugio de Vida Silvestre Texíguat (Fig. 7). These localities lie within the Premontane Wet Forest and peripherally in the Lower Montane Wet Forest formations of Holdridge (1967; as applied by McCranie and Wilson 2002).

#### Natural history.

The holotype was found coiled at 2130h approximately 2.5 m above the ground among old leaf sheaths in the crown of a medium-sized understory palm in gallery forest alongside the Río Jilamito (Fig. 4A). Anurans of the genera *Duellmanohyla* and *Ptychohyla* were abundant in the immediate vicinity of the holotype. Two adult males (CM 156870 and MVZ 269305) were collected along a ridge on the north side of La Liberacíon on the night of 28 July 2010. CM 156870 was active on a small tree from 0.5–1.5 m above the ground; the second snake (MVZ 269305) was sitting coiled on the ground at the edge of the trail, and attempted to escape by crawling across the path when we approached. In the immediate vicinity of these snakes were numerous *Craugastor rostralis* active on the ground and *Bolitoglossa* sp. active on low vegetation. Two neonates were collected on the same night (2100–2200 h) on 18 June 2010, and an adult female (USNM 579875) was collected the next night, in an area of elfin forest at 1,380 m on the ridge called Cerro El Chino (Fig. 4B) above the remote ranch locality La Liberación (at 1,030 m). The brown-phase neonate (USNM 579873) was found atop a large, similarly-colored dead palm frond, while the green-phase neonate (USNM 579874) was sitting in essentially the same ambush position as USNM 579873, but on top of a living green frond. Amphibian species collected in the immediate vicinity of *Bothriechis guifarroi* include *Bolitoglossa* sp., *Nototriton* sp., *Craugastor rostralis*, *Plectrohyla chrysopleura*, and *Ptychohyla spinipollex*. Twelve *Bolitoglossa* sp. were encountered the same night as the two neonates, all while active on or around dead and living palm fronds in the immediate vicinity of the neonates.

#### Remarks.

Townsend et al. (2012: 107) included a photograph of the holotype of *Bothriechis guifarroi* as “*Bothriechis marchi*.” *Bothriechis guifarroi* is typically distinguished from *Bothriechis marchi* by having the prelacunal scale fused to the second supralabial; however, one male paratype of *Bothriechis guifarroi* (CM 156870) has the right prelacunal separated from the second supralabial (they are fused on the left side). Also, one specimen of *Bothriechis marchi* (MCZ R-33335) from the “mountains west of San Pedro Sula” also has fused prelacunals and second supralabials on both sides, and another specimen (MCZ R-32030) from “La Cumbre” has the left prelacunal separated from the second supralabial (with them fused on the right side).

We tentatively refer four additional specimens to *Bothriechis guifarroi*: two from a locality on the leeward side of Refugio de Vida Silvestre Texíguat (USNM 337488–89, from 2.5 airline km NNE of La Fortuna, Dept. Yoro, 1,550 m elevation), one from the central portion of the Cordillera Nombre de Dios (USNM 319942, from Quebrada de Oro in Parque Nacional Pico Bonito, Dept. Atlántida, 1,090 m elevation), and one from “Tela” (AMNH 46949). All four specimens also have fused prelacunals and second supralabials on both sides. USNM 319942 was collected as a juvenile and raised in captivity (Wilson and McCranie 1992; McCranie 2011a), and exhibited a similar juvenile color pattern as USNM 579873 before undergoing an ontogenetic shift in coloration to the bright green pattern exhibited by the type series of *Bothriechis guifarroi*. Campbell and Lamar (2004: plates 385–386) also provided illustrations of juvenile *Bothriechis marchi* *s. l.* that demonstrated two color morphs similar to those exhibited by *Bothriechis guifarroi*; however these two individuals were captive born in the Houston Zoo and known only from “Honduras.”

AMNH 46949 was collected sometime during or before 1932 by Douglas March of the Lancetilla Serpentarium, just outside of the seaside city of Tela. While the “Tela” locality is considered erroneous (Wilson and McCranie 1992), it is possible that AMNH 46949 was obtained from somewhere in the nearby western portion of the Cordillera Nombre de Dios. Given that at least some highland taxa found at both RVS Texíguat and Parque Nacional Pico Bonito are endemic sister species (e.g., *Oedipina gephyra* and *Oedipina petiola*; McCranie and Townsend 2011), we refer USNM 319942 to *Bothriechis guifarroi* with the understanding that phylogenetic evaluation of the Pico Bonito population might eventually show those animals to represent a distinct taxon.

## Discussion

**Conservation status of *Bothriechis guifarroi*.** With the description of *Bothriechis guifarroi*, there are now at least three species of palm pitvipers endemic to the Chortís Highlands, including *Bothriechis marchi* and *Bothriechis thalassinus*, with the potential for additional undescribed taxonomic diversity pending phylogenetic evaluation of allopatric populations in central Honduras. Based on the IUCN Red List criteria (2012), *Bothriechis guifarroi* should be classified as Critically Endangered (B1ab[iii]+2ab[iii]) due to its limited known area of occurrence and the potential for anthropogenic damage to its habitat. According to the algorithm developed by Wilson and McCranie (2004a), we calculated the Environmental Vulnerability Score (EVS) for *Bothriechis guifarroi* as 5+8+5=18, allocating it to the category of a high vulnerability species. Given this conservation status, *Bothriechis guifarroi* becomes the 48^th^ member of the critically endangered endemic component of the Honduran herpetofauna (Wilson et al. 2012), and the tenth snake species and the first viperid species so designated. This species also warrants immediate consideration for protection under CITES, given its potential for exploitation in the pet trade.

The vicinity of the type locality of *Bothriechis guifarroi* is part of the relatively large and intact premontane rainforests and cloud forests of Refugio de Vida Silvestre Texíguat, one of the most important areas of herpetofaunal endemism in Mesoamerica (Townsend et al. 2012). While deforestation in the leeward portion of Refugio de Vida Silvestre Texíguat has been documented since at least the early 1990’s (see summary in Townsend et al. 2010), Townsend et al. (2012) reported that the windward portion of the reserve contained a large intact expanse of virtually undisturbed forest. In late 2012, a plot was cleared in the upper Río Jilamito watershed to the south of La Liberación, marking the first time farmers from adjacent Yoro had crossed into the Río Jilamito watershed and illegally cleared land (L. Herrera-B., pers. comm.). This is an ominous development, particularly in light of the recent drastic reduction in financial support for conservation efforts in Refugio de Vida Silvestre Texíguat, which had funded the training and employment of a team of local park guards during 2010–2012.

**Herpetofaunal Endemism in the Chortís Highlands.** Whereas Nuclear Central America has long been accepted as a region of high biodiversity and endemicity, some observers have further recognized the western and eastern portions of this highland block as distinct biogeographic entities (Johnson 1989; Campbell 1999; Townsend 2006, 2009). Eastern Nuclear Central America has been shown to have a distinctive component of endemic biodiversity, particularly in amphibians and reptiles (Wilson and Johnson 2010); however, molecular characterization of evolutionary diversification patterns in this region has been limited to a few studies of a restricted taxonomic breadth and broader geographic focus (e.g. Castoe et al. 2009). This region is geographically analogous to the Chortís Block, an allochthonous geological formation that currently forms the only modern continental portion of the Caribbean Tectonic Plate and the largest terrestrial segment of the contemporary Central American land bridge (Rogers 2003; Marshall 2007). The Chortís Block has a challengingly complex history, and recently has been the subject of increased focus, and sometimes contentious debate, within the geological research community (James 2007; Mann et al. 2007; Ortega-Gutiérrez et al. 2007; Silva-Romo 2008; Morán-Zenteno et al. 2009).

The majority of the geographical extent of the Chortís Highlands is found within the political boundaries of Honduras, the country with the highest degree of herpetofaunal endemism of any Central American nation (Wilson and Johnson 2010). Townsend and Wilson (2010) reported 91 endemic species (47 amphibians and 44 reptiles) from Honduras. Since that work went to press, an additional ten endemic species have been described from Honduras, including three new plethodontid salamanders (*Nototriton picucha*, Townsend et al. 2011; *Nototriton tomamorum*, Townsend et al. 2010; *Oedipina petiola*, McCranie and Townsend 2011), a new black iguana (*Ctenosaura praeocularis*, Hasbún and Köhler 2009), a new skink (*Marisora roatanae*; Hedges and Conn 2012), two new dwarf geckos (*Sphaerodactylus guanajae* and *Sphaerodactylus leonardovaldesi*; McCranie and Hedges 2012), and three new colubrid snakes (*Omoadiphas cannula*, McCranie and Cruz-Díaz 2011; *Tantilla psittaca*, McCranie 2011b; *Tantilla olympia*, Townsend et al. 2013). With these species included, the total stands at 101 species, making *Bothriechis guifarroi* the 102nd described herpetofaunal species endemic to Honduras.

***Bothriechismarchi* and the status of populations from Yoro.** We recognize *Bothriechis marchi* *sensu stricto* as occurring in localities in the Cordillera de Merendón in the Honduran departments of Cortés and Santa Bárbara along the border with Guatemala, as well as for at least one isolated locality in eastern Guatemala (Fig. 7). This Guatemalan locality, Cerro del Mono in Departamento de Zacapa, previously was the source of the only sequenced sample assigned to *Bothriechis marchi* (UTA R-52959; Castoe and Parkinson 2006). Although the population of *Bothriechis* from Cerro del Mono does not agree morphologically with the typical form of *Bothriechis marchi* (E.N. Smith, pers. comm.; see Plates 422–424 in Campbell and Lamar 2004, as *Bothriechis thalassinus*), the sequence data attributed to UTA R-52959 are not notably divergent from a sample of typical *Bothriechis marchi* (MVZ 263604) collected from the Sierra de Omoa in northern Honduras (Fig. 1).

The type localities of *Bothriechis marchi* and *Bothriechis thalassinus* are both in the Sierra de Caral, within approximately 20 km of one another on opposite sides of the Guatemala/Honduras border (Campbell and Lamar, 2004) suggesting that the two species occur either in parapatry or sympatry in the limited forest remaining in that mountain range (the type locality of *Bothriechis marchi* is not precise; Wilson and McCranie [1992] restricted it to the forested hills above El Oro, Departamento de Santa Bárbara). Individuals of both taxa in the Sierra de Caral exhibit a primarily green dorsal coloration, with some scattered bluish middorsal blotches (Campbell and Smith, 2000; Campbell and Lamar, 2004: plate 425). While the presence of *Bothriechis thalassinus* has not been confirmed by vouchered specimens from the Sierra de Omoa, the proximity of the Sierra de Caral and the Sierra de Omoa in northwestern Honduras and the similarity in coloration exhibited between these nominal taxa in that vicinity suggest the possibility that *Bothriechis thalassinus* may have gone unnoticed in the Sierra de Omoa. We have evaluated photographs of over a dozen individuals of green *Bothriechis* from the Sierra de Omoa encountered as part of an expedition-tourism operation in that area over the past five years, and have noted considerable variation in head scalation in the photographs. Unfortunately, none of the photographed individuals were collected nor were genetic samples taken to allow for more detailed evaluation of the *Bothriechis* of the Sierra de Omoa. In addition to the nominal form of *Bothriechis marchi*, it is likely that more than one species of *Bothriechis* occurs in sympatry or parapatry in the Sierra de Omoa, possibly including *Bothriechis thalassinus* and/or an unidentified sister taxon of *Bothriechis guifarroi*. Focused sampling and phylogenetic analysis of *Bothriechis* from the Sierra de Omoa is needed to better characterize the taxonomic diversity present in that mountain range.

Paraphyly in *Bothriechis marchi* *sensu lato* in terms of populations from the Cordillera de Merendón and the Cordillera Nombre de Dios, the latter now known to represent *Bothriechis guifarroi*, calls into question the taxonomic status of populations from isolated localities in the Sierra de Sulaco in Departmento de Yoro (Fig. 7). These populations are represented in collections by one specimen from Cerro de Pajarillos (USNM 561085), three specimens from the Montaña de Mataderos (FMNH 21777, MCZ R-38785–86), 14 specimens from “Portillo Grande” near Montaña Macuzal (FMNH 34732–35, 35895, 35999–601, 37217, 38542, 41621; MCZ R-38789–91), and two specimens from “Subirana Valley” (FMNH 21892, MCZ R-38788). Four of these specimens examined by us for this paper (MCZ R-38785–86, 38790–91) differ from *Bothriechis guifarroi* in having 21–19–15 dorsal scale rows (versus 19–19–15) and having varying conditions of fusion of the prelacunal and second supralabial (fused on both sides in MCZ R-38790, separate on both sides in MCZ R-38786, and fused on one side and separate on the other in MCZ R-38785 an R-38791). Given the considerable phylogenetic diversification presented by analysis of *Bothriechis guifarroi* and *Bothriechis marchi* *s. s.*, we cannot justify assignment of the Yoro populations to either taxon in the absence of molecular characterization of those populations. Therefore, we tentatively refer to the Yoro populations as *Bothriechis* species *inquirenda* pending collection of fresh material and phylogenetic characterization. Efforts to secure this material are currently underway.

**Evolutionary and biogeographic implications.** The phylogenetic position of *Bothriechis guifarroi* has significant implications for our understanding of the biogeography and evolution of palm-pitvipers. Crother et al. (1992) first presented a phylogenetic hypothesis for the genus *Bothriechis* supporting an eyelash pitviper clade (*Bothriechis schlegelii* and *Bothriechis supraciliaris*) that is sister to a clade containing the remaining species of *Bothriechis*, all of which have highland-associated distributions (typically <1,000 m elevation; Fig. 7). Castoe et al. (2009) and Daza et al. (2010) provided the most recent phylogenetic estimates for nine species of *Bothriechis*, as part of a comparative phylogeographic study of three co-distributed, predominantly Mesoamerican genera of pitvipers. Their phylogenetic hypotheses supported the contention of Castoe and Parkinson (2006) that *Bothriechis lateralis* is the sister group to the Nuclear Central American highland species of the genus (*Bothriechis aurifer*, *Bothriechis bicolor*, *Bothriechis marchi*, *Bothriechis rowleyi*, and *Bothriechis thalassinus*) and rejected the Crother et al. (1992) and Taggart et al. (2001) estimates that *Bothriechis lateralis* is the sister clade to *Bothriechis bicolor*. Our phylogenetic hypothesis also supports a Nuclear Central American clade, with *Bothriechis marchi* and *Bothriechis thalassinus* in the Chortís Highlands comprising a group that is sister to *Bothriechis aurifer*, *Bothriechis bicolor*, and *Bothriechis rowleyi* from the Nuclear Central American highlands to the west.

The Cordillera Nombre de Dios, which includesRefugio de Vida Silvestre Texíguat and Parque Nacional Pico Bonito, is home to a distinct endemic biota that, in many cases, bears little resemblance to the endemic communities found in other nearby cloud forests (Wilson and McCranie 2004b). While published phylogenetic datasets of taxa endemic to the Cordillera Nombre de Dios are limited, evidence is beginning to accumulate that this region, and Refugio de Vida Silvestre Texíguat in particular, represents a paleo-refugium where relict lineages have accumulated and persisted while disappearing elsewhere in northern Central America. *Bothriechis guifarroi* provides the best evidence to date in support of the paleo-refugium hypothesis, given its phylogenetic relationship with *Bothriechis lateralis* and *Bothriechis nigroviridis* from southern Central America and lack of close evolutionary relationships with other taxa from Nuclear Central America. Another recently described species from Refugio de Vida Silvestre Texíguat, the moss salamander *Nototriton tomamorum*, is morphologically and phylogenetically more closely associated with the Costa Rican taxon *Nototriton richardi* than it is with members of the northern *Nototriton barbouri* group, which includes an undescribed taxon that occurs in sympatry with *Nototriton tomamorum* (Townsend et al. 2010). *Isthmohyla insolita*, another Texíguat endemic, is one of only two *Isthmohyla* in northern Central America (the other being *Isthmohyla melacaena* from the Sierra de Omoa; neither species has been subjected to phylogenetic analysis), with the remaining 13 species restricted to highlands of Costa Rica and Panama (Köhler 2011).

Castoe et al. (2009) estimated that Lower Central American and Nuclear Central American *Bothriechis* diverged at the Nicaraguan Depression between 5.73–9.87 mybp, based on the hypothesis that *Bothriechis lateralis* represented the sister lineage to the Nuclear Central American clade. Given the discovery of *Bothriechis guifarroi*, the timing of divergence across the Nicaraguan Depression could be much more recent, similar to that seen between *Cerrophidion sasai* and *Cerrophidion wilsoni* (3.06–6.03 mybp; Castoe et al. 2009). Alternatively, it is possible that a Lower Central American *Bothriechis* (i.e., *Bothriechis guifarroi*, *Bothriechis lateralis*, *Bothriechis nigroviridis*) may have split from Nuclear Central American taxa via the Nicaraguan Depression earlier than suggested by Castoe et al. (2009) and Daza et al. (2010). Following additional sampling and the evaluation of populations presently referred to as *Bothriechis* sp. *inquirenda*, a reevaluation of Mesoamerican pitviper biogeography is warranted, and may shed further light on the complicated biogeographic relationship between northern and southern Central America.

## Supplementary Material

XML Treatment for Bothriechis guifarroi

## Appendix

**Specimens examined; museum acronyms follow Sabaj-Pérez (2012).**

*Bothriechis guifarroi* (12) – Honduras: ATLÁNTIDA: Quebrada de Oro, USNM 319942; “Tela,” AMNH 46949; La Liberación, CM 156870, MVZ 269305, USNM 579873–78, UTA R-60303. YORO: 2.5 airline km NNE of La Fortuna, USNM 337488–89.

*Bothriechis marchi* (19) – Honduras: CORTÉS: “near Cofradía,” MCZ 28014; “La Cumbre,” AMNH 46954–57, MCZ 32029–31; Sierra de Omoa, MCZ 33334–36, 33560–64, USNM 83454. SANTA BÁRBARA: “Quimistán,” MCZ 27260, 27510; “between Cofradía and Quimistán,” MCZ 27567–68, UMMZ 90677 (was MCZ 27569); “Santa Bárbara,” MCZ 28014.

*Bothriechis thalassinus* (3) – Honduras: COPÁN: Quebrada Grande, KU 203094; OCOTEPEQUE: 21.7 km east of Nuevo Ocotepeque, LSUMZ 23821; SANTA BÁRBARA: southeastern slope of Cerro Santa Barbara, LSUMZ 11638.

*Bothriechis* sp. *inquirenda* (4) – Honduras: YORO: Montañas de Mataderos, MCZ 38785–86; Portillo Grande, MCZ R-38790–91.
